# Novel Program Connects Medical Students with Startups Focused on Social Determinants of Health

**DOI:** 10.1007/s11606-024-08942-0

**Published:** 2024-07-29

**Authors:** Danielle E. Brown, Angela Malinovitch, Hannah Posner, Deepak Indrakanti, Medha Sharma, Michael Karamardian, C. Jessica Dine

**Affiliations:** Perelman School of Medicine, University of Pennsylvania, Philadelphia, PA USA

## Abstract

**Background:**

Understanding health equity is critical for the development of patient-centered physicians, but few avenues exist for medical students to participate in experiential learning related to social determinants of health (SDOH).

**Aim:**

To create and evaluate the PennHealthX SDOH Accelerator Program, which pairs students with health equity startups.

**Setting:**

The program matches medical students at our institution with startups focused on SDOH for voluntary, part-time internships.

**Participants:**

Medical students of all years are eligible to apply. Startups are typically early-stage (≤ 10 employees).

**Program Description:**

Two pre-clinical students lead the program. Startups are sourced via alumni networking, partnerships with venture capital firms, and cold outreach. Startups and students apply and are matched based on project goals and student backgrounds/skills. Upon completion, feedback is gathered through open-ended interviews with all students.

**Program Evaluation:**

Twenty medical students were matched with 11 startups. In post-program interviews, students expressed high satisfaction. Students specifically valued the opportunity to learn about SDOH in a hands-on and solution-oriented way.

**Discussion:**

This program gives students the opportunity to impact their communities and learn about addressing SDOH with innovative solutions. We are continuing to build the program at our institution and expand its impact to other medical schools.

**Supplementary Information:**

The online version contains supplementary material available at 10.1007/s11606-024-08942-0.

## INTRODUCTION

Social determinants of health (SDOH) are the factors that shape health prospects beyond genetics and medical care, including an individual’s social circumstances, environment, and behaviors.^1^ There has been growing recognition of the role that SDOHs play in health outcomes and a push for physicians to consider these circumstances when treating patients.

It is critical for student-doctors to learn about SDOH during their undergraduate medical education. Currently, medical students engage with SDOH predominantly in three ways: curricular didactics, academic research, and community service. Over 85% of medical students report exposure to SDOH through their core curriculum, often during the pre-clinical year(s).^2^ However, medical schools report barriers to integrating SDOH into curricula, including lack of faculty availability and limited space in curriculum.^3^ Additionally, there is potential benefit to incorporating SDOH into medical school education through an experiential learning approach, where students can integrate themselves into an environment to “learn by doing” and then reflect on their work.^4^

As focus on health equity has increased worldwide^5^, many startup companies have formed to create products and services addressing SDOH and access to care. The approaches taken by startup companies to solve these problems differ from academic research, given their differing stakeholders, funding sources, and professional perspectives. Medical students rarely have opportunities to engage with health equity topics through the lens of entrepreneurship, which offers a fast-paced environment for students to dive deeply into a specific health equity topic over a short time period. Conflicting incentives can arise for healthcare professionals engaged in SDOH entrepreneurship, but we believe physicians and trainees can take on the important role of patient advocacy in these settings.

Therefore, we created the PennHealthX SDOH Accelerator Program at the Perelman School of Medicine. This program is sponsored by PennHealthX, a student-run group focused on the intersection of medicine, business, and technology. It is the first program of its kind nationally and gives medical students the opportunity to work directly with SDOH startup companies. We believe this program provides students with an understanding of SDOH that is complementary to their curriculum, enhances their education through experiential learning, and furthers their development into well-rounded clinicians.

## SETTING AND PARTICIPANTS

The SDOH Accelerator Program has two aims. First, we aim to provide medical students with the opportunity to deepen their understanding of social determinants of health through hands-on experiences and further develop problem-solving skills. Second, we aim to address healthcare inequities through partnerships with health equity–focused startups. To accomplish these aims, the program matches medical students with startups focused on SDOH for voluntary, part-time internships. Startups are early-stage companies (1–10 employees), which allows students to work directly with founders. When determining startup eligibility, SDOHs are broadly defined. Any startup that improves care for underserved populations, addresses non-medical factors that impact health outcomes, or expands access to healthcare services is eligible. Students work 5–10 h per week for 4 months and receive a $1000 stipend funded by the PennHealthX endowment. Students in all years of medical school are eligible to participate, including “year-out,” dual degree, and PhD students.

## PROGRAM DESCRIPTION

**Figure 1:**
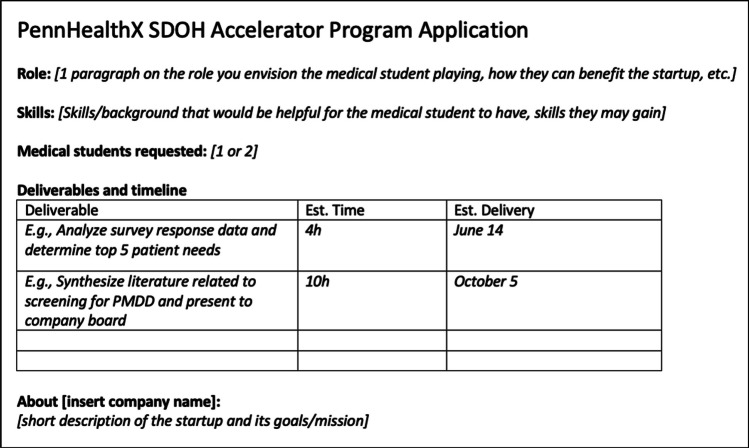
Startup company application and work plan template.

The SDOH Accelerator Program is led by two pre-clerkship medical students who execute two major program components: (1) sourcing and vetting companies and student applications and (2) actively managing student projects. In the development and evaluation of the program, we employed a logic model. This logic model is a systematic visual representation that outlines the relationships between our program’s resources, activities, outputs, and desired outcomes, which provides a structured way to plan, implement, and evaluate the program. Similar to the Plan-Do-Study-Act (PDSA) cycle, this approach allows us to continuously refine and improve the program based on feedback from participants and observed outcomes, as described below. Originally, companies were invited through cold email outreach, but this approach led to suboptimal startup investment in the program, as some companies passively agreed to participate without assessing their capacity to mentor a student. Therefore, we introduced an application process to better evaluate each startup’s commitment to the program. This application process includes submitting a workplan (Fig. 1) and interviewing with program leaders to discuss the company’s vision for a student consultant. If determined eligible, this meeting also allows startups to communicate their desired number of medical students and any requests for specific backgrounds/skills. To source students, an application is distributed to the medical student body, with a request for students to submit their resume and answer short essay prompts (Supplemental Figure). Candidates are selected based on relevant skillsets for the specific project and their articulated passion for learning about SDOH and entrepreneurship in their responses. Originally, the 4-month internships aligned with the academic semester (September–December or January–April). However, program timing became more flexible when students and companies voiced preference for individualized timing based on their schedules.

After company and student selection, program leaders begin their management of the student-company relationship. Originally, the role of the program leaders was finished after initiating student-founder introductions. However, we discovered this approach lacked adequate accountability for both the startup and student consultant(s). Therefore, program leaders now host introductory meetings between the student consultant(s) and startup liaisons, where all participants review the preliminary work plan and timeline together. We have also implemented a check-in process. Student consultants send monthly progress updates to the program leaders, which allows for early detection of any roadblocks and the opportunity for intervention if necessary. The program leaders cannot participate as interns during their leadership term, as their role involves oversight of all projects.

Over the first 2 years, our model for sourcing companies has also evolved. Originally, we attempted an advertising campaign, with the rationale that companies who approached us after seeing our materials would be more invested in the program. We published articles in our health system’s magazine, sent out an alumni newsletter, and created a website with the program description and company application. However, these materials were not reaching the intended audience of startup founders. Therefore, we pivoted to partnering with startup-adjacent organizations (e.g., venture capital firms) to pitch the program directly to their startup portfolios. Throughout this time, we continued cold email outreach to startups in our area that we found through online research, which also resulted in multiple participating companies. See Table 1 for participating company descriptions.

**Table 1:** Characteristics of Startup Companies and Student Projects

Company focus	Location	Student deliverable
App tackling racial disparities in maternal health outcomes via knowledge-sharing	Durham, NC	• Prepared applications for NIH, academic medical center, and foundation grants • Assisted in limited release (beta launch) of first digital app
Creation of sustainable sourcing model for excess food waste	Philadelphia, PA	• Created model depicting components of supply/distribution market for excess food
Healthy food access in low-income neighborhoods through hydroponic farming	Philadelphia, PA	• Developed presentation for founder to use with potential investors • Researched community partners involved in expanding hydroponic farming access
Mobile app to combat misinformation among parents of pediatric patients	Raleigh, NC	• Connected founders to pediatricians • Edited marketing materials to resonate with clinician-based audiences
Cold therapy wearables and women’s health education	San Francisco, CA	• Translated research articles into digestible, patient-friendly articles • Worked with a company illustrator to create educational graphics
Mobile healthcare and health insurance registration for low-income individuals at laundromats	Philadelphia, PA	• Identified greatest unmet health needs of patients at laundromats • Created a database of local healthcare organizations for patients to reference
Tech-enabled diabetes prevention for patients without primary care access	Philadelphia, PA	• Identified wellness centers that provide diabetes prevention education to serve as potential collaborators • Identified and assessed investors for the first level of funding (Series A) based on investment focus and mission
At-home lung monitoring for asthma patients	Philadelphia, PA	• Created script for investors and physicians about lack of effective continuous respiratory monitoring • Synthesized clinical trial data
Women’s health empowerment and education for hormonal disorders	Montreal, QC	• Conducted literature review based on research team ad hoc clinical and scientific questions
At-home and high-quality STI testing	Lausanne, Switzerland	• Compiled data about number and location of LGBTQ + clinics in the United States • Created a discussion guide to inform primary research interviews
Integrating SDOH module into oncology care and analytics platform	Philadelphia, PA	• Implemented an SDOH screening tool into company’s oncology data collection platform

Abbreviations: SDOH, social determinants of health. STI, sexually transmitted infection. Note: only includes participating companies prior to December 11, 2023

At the conclusion of each internship, we gather feedback about the program from student consultant(s) through interviews and/or feedback forms. All students are interviewed by program leaders using a standardized interview guide. Interviews are 20–30 min in duration and cover four topics: the final status of student deliverables, aspects of the process that went well, aspects that could be improved, and any additions to the program if time/budget were not constraints. This year, we are in the process of conducting an IRB-exempt research study to formally analyze student experiences. Finally, an annual SDOH Accelerator symposium is held, where each student consultant presents their work to other program participants, medical student peers, and faculty.

## PROGRAM EVALUATION

Overall, 20 medical students have been matched with 11 companies. Ninety percent of students worked in pairs, given feedback that working together enhanced the experience and productivity of the student consultants. By gender, 14 students were female and six male. The majority of students (60%) identify as non-white. There were 13 pre-clerkship, two clerkship, and five post-clerkship students. The cohort had experience with different industries prior to medical school, including management consulting, journalism, product development, digital health, and software engineering. Ten students (50%) had full-time work experience, which is roughly representative of our student body.

The 11 participating startups address numerous SDOHs in innovative and technology-driven ways (Table 1). For example, one company promotes knowledge-sharing among Black women during pregnancy through a cellphone app. Another company partners with insurance providers to bring preventative healthcare services to low-income individuals at laundromats. Each student’s project was customized to align with the startup’s goals and the student’s skills (Table 1). For example, two students translated clinical literature about the company’s product into digestible patient-education articles. Another student interviewed individuals at laundromats to understand their health-related needs and worked with clinicians at our university to compile relevant local resources.

While reflecting on their experiences in post-program interviews, students reported high satisfaction. Formal analysis of interview responses was not conducted, given the purpose of interviews was program evaluation to modify program structure in real time. Students often highlighted two common themes: learning to apply theoretical knowledge about SDOH in real-word scenarios and increasing their proficiency in practical skills (e.g., Excel, PowerPoint). Examples of skills cited by students included applying evidence-based medicine in the business environment and working independently on a discrete project while on a broader team.

Ultimately, the SDOH Accelerator Program met its goal of successfully matching students interested in social determinants of health with startups focused on health equity. Its value is bidirectional — students gain exposure to tackling SDOH within the entrepreneurial space and develop professional skills applicable to their future careers, while social impact–focused startups gain voluntary collaboration with students who bring knowledge and clinical perspectives.

## DISCUSSION

SDOHs play a critical role in health outcomes in the United States. National organizations, such as the American College of Physicians and the National Academy of Sciences, advocate that future physicians should learn about patients’ social risk factors in order to effectively treat patients. The SDOH Accelerator Program is an innovative, experiential-based program that can teach medical students about how individuals, companies, and communities are working to tackle inequities and empowers students to join the effort.

Undergraduate medical education could benefit from greater integration of SDOHs into the medical student experience. Current exposure primarily involves didactic teaching, academic research, and community-service opportunities. Although important, these approaches sometimes leave students and physicians feeling powerless to address the systemic causes of SDOH that they see impacting their patients. The SDOH Accelerator Program provides the opportunity for students to learn about SDOH in a hands-on, solution-oriented way.

Examining how this program could fit into undergraduate medical education can be understood through the framework of an experiential learning model. This framework advocates that education happens best when learners immerse themselves into the context of a topic and connect what they learned to future implications. The model includes four components: concrete experience, reflective experience, abstract conceptualization, and active experimentation.^6^ The SDOH Accelerator Program is a concrete experience for medical students that gives them exposure to what these problems and solutions look like in practice, allowing them to also try experimentation. In future years, to further the experiential learning approach, we plan to include intentional reflection by asking program participants to consider how they will incorporate this experience into their future careers as physicians.

There are limitations to the program’s innovative design and potential to scale. First, students self-select into the program, leading to a cohort that overrepresents students with backgrounds in business and health systems research. The selection process aims to identify candidates with skills for the specific role, as well as candidates who are seeking exposure to these topics. Currently, there is no formal mechanism to reduce bias in our process, which will be an important addition going forward. Second, given the decentralized nature of each partnership, students have heterogeneous experiences in health equity topic exposure and gain variable skills. However, this design allows us to tailor each project to student interests and company needs. Third, the program does not currently have formal programming about the potential ethical dilemmas of trainee involvement with startups, which are often for-profit entities. Future curricula will be developed to include discussions about these nuanced topics. We believe physician involvement in healthcare entrepreneurship helps amplify patient voices in settings where they are often overlooked. All program startups were selected based on a mission or product addressing unmet needs for vulnerable populations. Finally, the program does require monetary support from medical school administration to fund student stipends.

Our experience creating and leading the SDOH Accelerator highlighted key learnings for other medical schools interested in creating similar programs. We believe this model would be valuable to any medical school seeking to augment its SDOH education, especially through experiential, solution-oriented opportunities.

## Supplementary Information

Below is the link to the electronic supplementary material.

Supplementary file1 (DOCX 76 KB)

## References

[1] (AAHC) AoAHC. Academic Health Centers and the Social Determinants of Health: Challenges & Barriers, Responses & Solutions. 2015.

[2] Colleges AoAM. Curriculum Inventory: Social Determinants for Health by Academic Level. 2019.

[3] **Lewis JH, Lage OG, Grant BK, et al.** Addressing the Social Determinants of Health in Undergraduate Medical Education Curricula: A Survey Report. Adv Med Educ Pract. 2020;11:369-377. 10.2147/amep.S243827 32547288 10.2147/AMEP.S243827 PMC7250290

[4] **Yardley S, Teunissen PW, Dornan T.** Experiential learning: transforming theory into practice. Med Teach. 2012;34(2):161-4. 10.3109/0142159x.2012.643264 22288996 10.3109/0142159X.2012.643264

[5] **Yao Q, Li X, Luo F, Yang L, Liu C, Sun J.** The historical roots and seminal research on health equity: a referenced publication year spectroscopy (RPYS) analysis. Int J Equity Health. 2019;18(1):152. 10.1186/s12939-019-1058-3 31615528 10.1186/s12939-019-1058-3 PMC6792226

[6] **Yardley S, Teunissen PW, Dornan T.** Experiential learning: AMEE Guide No. 63. Med Teach. 2012;34(2):e102-15. 10.3109/0142159x.2012.650741 22289008 10.3109/0142159X.2012.650741

