# Supplementary figures and images for: Novel Program Connects Medical Students with Startups Focused on Social Determinants of Health
Source: J Gen Intern Med. 2024 Jul 29;40(2):474–8. doi: 10.1007/s11606-024-08942-0 (PMC11803028; doi:10.1007/s11606-024-08942-0)

**SUPPLEMENT**

Supplemental Figure: Medical Student Consultant Application


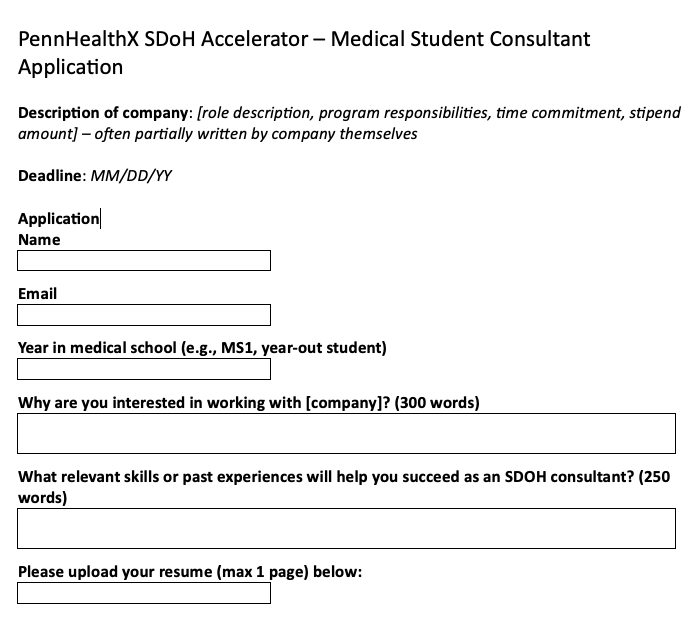

Supplement: Supplementary file 1 — Supplementary file1 (DOCX 76 KB) [file 11606_2024_8942_MOESM1_ESM.docx]
